# Screening for endogenous hypercortisolism in patients with osteoporosis and fractures: why, when and how

**DOI:** 10.1007/s40618-024-02450-y

**Published:** 2024-10-03

**Authors:** Roberta Giordano, Mirko Parasiliti Caprino, Paola Loli, Andrea Giustina

**Affiliations:** 1 Department of Biological and Clinical Sciences, University of Turin, Turin, Italy; 2 Division of Endocrinology, Diabetology and Metabolism, Department of Medical Sciences, University of Turin, Turin, Italy; 3 Institute of Endocrine and Metabolic Sciences (IEMS), Università Vita-Salute San Raffaele IRCCS Ospedale San Raffaele, Via Olgettina, 58, Milano, 20132 Italy

**Keywords:** Cushing’s syndrome, Osteoporosis, Vertebral fractures, Bone mineral density, Vertebral morphometry, Glucocorticoids, Screening

## Abstract

Skeletal comorbidities are frequent and clinically relevant findings in Cushing’s syndrome (CS) since an uncoupled suppressed bone formation and enhanced bone resorption leads to a marked skeletal damage with a rapid increase of fracture risk. Reduced Bone Mineral Density (BMD) has been consistently reported and osteopenia or osteoporosis are typical findings in patients with CS. Vertebral Fractures (VFs) are frequently reported and may occur even in patients with an only mild reduction of BMD. Since CS is diagnosed late due to often difficult biochemical and radiological confirmation as well as to signs and symptoms common in other much more frequent diseases an approach suggested for overcoming underdiagnosis is to screen patients with manifestations which may overlap with those of CS such as arterial hypertension, diabetes mellitus and osteoporosis. Our review will focus on the rationale and best practice for screening osteoporotic patients for CS.

## Introduction

The negative effects of endogenous glucocorticoid excess (Cushing’s syndrome, CS) are well known [1]. In fact, Cushing’s disease (CD), caused by a pituitary ACTH adenoma and the most frequent form of endogenous hypercortisolism, increases mortality and decreases quality of life (QoL) leading to severe systemic comorbidities [2], including osteopathy, which is the most severe form of secondary osteoporosis and may occur early in natural history of CD [3], as recently reported also for other pituitary secreting adenomas [4]. Moreover, in adrenal forms of CS bone damage may even be greater due to the absence of hyperandrogenism which may be somewhat protective against the glucocorticoid-induced osteopathy of CD [5]. Therefore, accurate diagnosis and management of CS and its complications are required to improve patient outcomes [6]. However, unfortunately, diagnosis of CS is often delayed for long time, on one side due to lack of knowledge in the medical community about this insidious and progessive disease process and on the other side by complexity and not particularly easy interpretation of baseline and dynamic hormonal tests [7].

Based on the widespread involvement of bone in CS, which is often characterized by misdiagnosis, diagnostic delay and underdiagnosis [7], several authors have proposed that for solving these clinical issues at least in part bone clinics could be good candidates for performing an effective CS screening [8]. In this narrative review we aimed at evaluating the evidences for the utility of screening for CS in patients with osteoporosis and fracture and suggest indications on why, when and how CS could be screened in the bone clinic.

### Bone in CS: pathophysiological and clinical aspects

#### Pathophysiological aspects

Chronic glucocorticoid excess heavily impacts on the cellular phase of bone metabolism by suppressing bone formation by directly impairing osteoblast differentiation and enhancing apoptosis of osteoblasts and osteocytes [9]. Moreover, bone resorption is initially increased due to early enhancement of osteoclast function, maturation and differentiation [10], which in turn leads to uncoupling of bone turn-over with reduced formation and increased resorption [11]. As a result, a rapid increase of fracture risk is observed in glucocorticoid excess linked more to impaired microarchitectural properties of bone than to a reduced bone density [12]. Moreover, excess glucocorticoids may negatively impact skeletal health through many indirect mechanisms. In fact, they may cause hypogonadism in both males and females, impairing both gonadal steroids and gonadotrophin secretion [1] and hyposomatotropism through an increase in hypothalamic somatostatin secretion [13, 14]. They may also reduce insulin-like growth factor-1 (IGF-I) secretion and action [15]. Inasmuch, glucocorticoids may impact also on the mineralization phase of bone metabolism by interfering with vitamin D metabolism with resulting vitamin D deficiency [16, 17] and activation of cholecalciferol into 25-OH VD with reduced expression of vitamin D receptor in several tissues [16, 17]. Furthermore, glucocorticoids stimulate parathyroid hormone pulsatile secretion phase possibly leading to secondary hyperparathyroidism [18]. Finally, sarcopenia and obesity are particularly frequent comorbidities of CS and increasing the risk of falls could contribute, consequently, to a higher risk of fragility fracture [19].

#### Clinical aspects

It is well known that measurement of bone mineral density (BMD) by Dual-energy X-ray Absorptiometry (DXA) is the gold standard for diagnosis of primary forms of osteoporosis, accounting for more than two thirds of the whole bone strength [20].

Independently from the underlying cause (pituitary, adrenal or ectopic), a decrease in BMD (about 20% vs. healthy subjects) was consistently reported in CS and osteopenia (T-score between − 1.0 and − 2.5DS) or osteoporosis (T-score ≤-2.5DS) are frequently found in CS patients [21]. Densitometric osteoporosis was reported in 28–50% of CS patients and it was not associated with age, disease duration and other associated comorbidities such as diabetes or hypogonadism [21–23] whereas males with CD were reported to have more frequently lumbar osteoporosis than females [24]. Thus, hypercortisolism, also in patients with mild autonomous cortisol secretion (MACS) [25], may be associated with early bone damage independently from the coexistence of other possible risk factors [26, 27].

In agreement with the notion that trabecular bone is the most impacted by glucocorticoids, lumbar spine BMD is decreased more than forearm and hip BMD [28] as confirmed by high-resolution peripheral quantitative computed tomography (HRpQCT) which also suggested that in CS reduced cortical thickness and increased cortical porosity may coexist [29]. Also trabecular bone score (TBS), another index of bone quality which is derived from lumbar DXA scans, may be impaired in CS and may predict according to some studies fragility fractures risk more efficiently than BMD alone [30]. Finally, bone quality can be also indirectly assessed by assaying circulating markers of bone turnover, such as osteocalcin which is a marker of bone formation and is heavily decreased in states of hypercortisolism also predicting the fracture risk [29, 31].

The predominant bone quality defect leads to the occurrence of fragility fractures in situations of glucocorticoid excess even in the presence of mild or no reduction in BMD (normal or osteopenic densitometric state) [32]. Importantly, fractures could be the presenting or, due to their severity and number, the predominant manifestation in some CS patients [32]. Prevalence of osteoporotic fractures is highly variable in different studies reaching in some the 50% [1] with the majority of them reporting thoracic and lumbar vertebral fractures with no gender differences and only up to 15% of CS patients experiencing hip, wrist and humerus non-traumatic fractures [16]. Vertebral fractures are still under- or mis -diagnosed, as they are often asymptomatic. Therefore, as in other forms of secondary osteoporosis [33–35], the morphometric evaluation became key for a correct assessment of vertebral fractures which through this approach may be found in up to 78% CS patients [36]. Recently, post-menopausal women with MACS were reported to bear an high risk of vertebral fractures [37]. Interestingly, a novel approach combining DXA and vertebral morphometry has been recently proposed for diagnosis and follow-up of bone comorbidity [38] in CS. Association between osteoporosis or vertebral fractures and cortisol levels is still unclear. Most of the studies suggested that cortisol values may predict fracture risk based on the observation that multiple and severe fractures are more frequently found in patients with ectopic CS with respect to those with CD and likely lower cortisol levels [38].

### Epidemiology of CS in patients with osteoporosis and fractures

In a paper from the German registry, among patients referred to the Endocrine clinic for clinically suspected CS osteoporosis was one of the main complaints (8% vs. 2%; *P* = .02) in patients with confirmed vs. non-confirmed diagnosis of CS [39].

MACS, a condition of impaired hypothalamic-adrenal-axis homeostasis without the classical signs and symptoms of glucocorticoid excess, is a recently defined entity, which has been shown to be associated to increased bone resorption, bone loss, and high prevalence of vertebral fractures regardless of gonadal status [40]. In particular, the prevalence of MACS in patients with osteoporosis may be underestimated, as reported by a cross-sectional study conducted in a large group of patients with osteoporosis [8]. The prevalence of MACS in subjects referred for osteoporosis has been mainly assessed in cross-sectional studies or post hoc analyses and varies according to different reports from 0.6 to 3.8% being even higher in fractured subjects (1.9 to 17.6%) [41]. Prospective large controlled studies have been advocated in order to confirm these data, allow evaluation of the natural history of skeletal comorbidity, establish more accurately prevalence of hypercortisolism and respective efficacy of bone-protective vs. tumor-targeted treatments [42].

### Patient selection and tools for screening of CS in patients with osteoporosis

#### Patient selection

Based on 2008 Endocrine Society guidelines, adult patients with unusual symptoms for their age (such as osteoporosis or hypertension), with multiple and progressive hypercortisolism-linked symptoms should be screened for CS [39, 43].

Screening programs as defined by world health organization (WHO) should be implemented in an asymptomatic phase of a disease [44]. Whereas mild hypercortisolism may fit in this definition it has to be noted that often clinical manifestation of CS is overlooked by many physicians [2]. Therefore, in such cases bone evaluation can not be properly defined a screening test. In this regard it is of paramount importance the accurate collection of patient history in the DXA laboratory which is clearly more likely to be effective when this is run in such a context by endocrinologists.

Correctly defined screening thus applies to truly asymptomatic mild hypercortisolism [45]. As previously reported in this context not all WHO guidelines for establishing screening programs are yet met [42, 44]. Therefore, several authors attempted to better define the criteria for selection of patients in whom screening for CS in bone clinics may be more effective than a wait and see approach such as young patients, men and pre-menopausal women with low vertebral bone mass and fractures [46]. Unfortunately, it is not well established if we should exclude CS in the general population affected by fragility fractures [44]. Nevertheless, some patients with occult endogenous CS with osteoporosis and fractures as the sole manifestations of the disease have been reported [23]. Thus, it has been suggested that hypercortisolism should be ruled out in patients in whom the etiology of osteoporosis is not readily apparent.

#### Tools for bone health evaluation

Based on what we know on the effects of hypercortisolism on measures of bone health status the choice of the tool(s) to be used for the screening of CS in the bone clinic needs to be carefully discussed.

In fact, in patients with CS the evaluation of fracture risk, using FRAX tool, is not validated and the investigation of bone complications through DXA may not have satisfactory accuracy [47]. The evaluation of bone quality with trabecular bone score a DXA derived parameter may be of help since glucocorticoids are known to prevalently affect bone quality vs. bone quantity [29]. Other more sophisticated measures of bone quality such as HRpQCT are not yet widely available and can not be suggested for screening purposes also for their cost [29]. The morphometric vertebral assessment appears to be currently the most effective screening approach for CS since trabecular bone is the most affected by hypercortisolism, clinical vertebral fractures are largely underestimated and it can be integrated in DXA measurements but can also be opportunistically applied to chest X-rays [48]. Finally, biochemical assessment of bone markers such as osteocalcin which is suppressed in hypercortisolism may be a useful complement to bone evaluation further refining the patient selection of patients to be screened for CS in the bone clinic [29, 31]. (Table 1).

**Table 1:** Role of different methods of bone health assessment in screening of CS

	BMD	BTM	Morphometry	Bone quality
Asymptomatic	**↑**	**↑↑**	**↑↑↑**	**↑↑**
Symptomatic	**↑**	↑**↑↑**	**↑↑↑**	**↑**

BMD, bone mineral density; BTM, bone turn-over markers

### How to biochemically screen CS in osteoporotic/fractured patients

One of the main general limitations of screening for MACS consists in the uncertainty about the method(s) to use for validating its diagnosis since in absence of specific clinical features results of dynamic tests may overlap between patients with or without hypercortisolism [49] and a gold standard is not yet available [42].

A specific limitation for CS screening in bone centers is that no clear data are available on how the biochemical screening for CS should be performed in patients with bone derangement. In fact, in the literature, there are not specific indications about what should be the preferred test for the diagnosis of CS in this group of patients [42].

As indicated in international guidelines, the laboratory tests most commonly used for CS diagnosis are 24-hour urine-free cortisol (UFC), serum cortisol after low-dose (1 mg) dexamethasone suppression test (DST) and late-night salivary cortisol (LNSC) [2, 43]. In this setting, sensitivity of all tests is higher than 90%; the highest sensitivity rates are obtained with DST and LNSC and the lowest with UFC. Specificity is somewhat lower than sensitivity, with LNSC being the most specific and DST and UFC the least specific [2, 43, 50].

In the last international guidelines [2], there is no single preferred diagnostic test for CS, and there is not consensus on how to decide whether and when to test, despite attempts to develop a score for ease of diagnosis. Clinical judgment and index of suspicion for CS are important and underscore the need to individualize decisions about timing and selection for diagnostic testing on the basis of the clinical scenario [51]. In fact, both accurate history collection and detailed physical exam in order to detect the physical stigmata of CS among patients referred to bone clinic should have a key role and it may be considered to specifically train ad hoc bone experts at least in some more specialized centers [52]. If CS is suspected, any of the above-mentioned diagnostic tests could be useful.

LNSC has recently gained popularity due to its high diagnostic sensitivity and specificity, noninvasive sample collection, and cost effectiveness [2, 53, 54]; it should be preferred in patients with renal impairment (creatinine clearance < 60 mL/min) or clinically significant polyuria (> 5 L/24 h), because urine volume and glomerular filtration rate strongly predict UFC; by contrast, LNSC should not be done in patients with disruption of the normal day and night cycle, such as night-shift workers. Patients with mild CS may have LNSC just above the upper limit of normal (ULN) [54, 55] but LNSC may perform better than UFC in diagnosing mild forms of CS [2, 56].

Regarding UFC, at least two or three 24-h urine collections are advised to account for intra-patient variability. The advantage of UFC over DST is that overall cortisol production is independent of corticosteroid binding globulin (CBG) changes and dexamethasone metabolism or compliance. Sex, body-mass index (BMI), age, very high or low urinary volume, impaired renal function and sodium intake can all influence UFC concentrations and should be taken into account for interpretation [2].

The third strategy of case finding for CS is represented by DST, [56]. The pitfalls of this method are the possibility of false positive results in patients with rapid absorption or malabsorption of dexamethasone due to increased gut transit time (as in celiac disease or chronic diarrhea); in concomitant treatment with CYP3A4 inducers (such as phenobarbital, carbamazepine and St John’s wort); and in increased CBG concentrations caused by oral estrogens, pregnancy, or chronic active hepatitis, which can increase total cortisol concentrations [2]. The measurement of dexamethasone with cortisol may reduce the risk for false-positive results [57]. As well, either LNSC or UFC may be used to enhance the diagnostic accuracy of an unsuppressed DST by reducing false-positive test results [58, 59], False-negative results are less common, typically resulting from inhibition of dexamethasone metabolism by concomitant medications (cimetidine, fluoxetine or diltiazem), leading to a higher biologically available dose. Decreased CBG and albumin concentrations, for example in patients with nephrotic syndrome, also might produce falsely low values [60].

In patients with bone derangement there is no single preferred screening test for CS. Clearly, for a screening method in principle a test with the highest sensitivity vs. specificity should be chosen. In this regard, also considering its convenience, assessment of LNSC appears to be the best candidate method although DST was the most frequently used so far in literature [60] particularly in the suspicion of adrenal CS, also because LNSC measurement is still not widely available (Table 2).

**Table 2:** Role of different methods of biochemical assessment in screening of CS

	UFC	LNSC	DST
Asymptomatic	**↑↑**	**↑**	**↑↑↑**
Symptomatic	**↑↑↑**	↑**↑↑**	**↑↑↑**

DST, Dexamethasone suppression test; LNSC, Late night salivary cortisol; UFC, Urinary free cortisol

### **CS screening in osteoporotic/fractured patients: why**,** when and how**

#### Why

Skeletal comorbidities are frequent and clinically relevant findings in CS. Infact, vertebral fractures, the landmark of the bone involvement, may occur early in the history of the disease [61]. Unfortunatley, CS is still largely underdiagnosed and misdiagnosed with resulting long diagnostic delay which in turn has relevant implications in prognosis, treatment and quality of life of the patients [62, 63]. Screening for CS in bone centers is part of an effort of improving and anticipating the diagnosis of the disease which also involves cardiologists, diabetologists and psychiatrists [64–66] in the vein of what has already been suggested for other pituitary conditions such as acromegaly characterized by relevant diagnostic delay [67].

#### When

##### Clinically manifest but misdiagnosed CS

Clinical diagnosis of CS is made difficult by the presence of signs and symptoms that are common in the general population such as obesity, diabetes, osteoporosis and depression [2]. Moreover, not all these manifestations may have the same severity in each single Cushing patient [68]. Therefore, particularly early diagnosis is uncommon outside the Pituitary Tumors Centers of Excellence (PTCOEs) or specialized centers among general physicians [69].

“Non classical” screening in this context is represented by a diagnosis of CS made in the bone centers in patients with already clear cut coexisting manifestations of CS. In this context the presence of a bone expert in PTCOEs is recommended [70]. Moreover, the accuracy of the patient history collection with particular attention to resistant hypertension, (recent) increase in body weight and glucose intolerance or diagnosis of depression [71] should be proactively looked at in the bone clinic and items concerning these issues should be included in the clinical questionnaires filled by the patients in the DXA laboratory. It occurs at a more advanced stage of disease but at least represents the entry door of the Cushing patient in the endocrine care system.

##### Asymptomatic mild hypercortisolism

In this setting bone evaluation is a “ classical” screening test which suffers from all the cost/effectiveness implications of all other screenings [44]. It represents an attractive chance to diagnose CS in its early stages but it is limited by uncertainties in patient selection and more appropriate bone tools for realizing a cost/effective screening [36]. In line with what proposed in literature it can be suggested to proactively search for hypercortisolism in 1. patients with densitometric diagnosis of osteoporosis in whom (a) BMD is reduced as compared to age- matched controls (Z score value < -2 SD), (b) BMD declines more rapidly than expected, (c) suboptimal densitometric response to bone protective therapy is observed and 2. Patients undergoing fragility fractures with uncommon features such as (a) young (< 50 years old) men and pre-menopausal females; (b) eugonadal subjects; (c) DXA BMD values inappropriately in normal range for age or only slightly reduced (in fact glucocorticoids impair largely more bone quality than quantity) [11].

### How

#### Biochemical tests

No guidelines exist towards the best biochemical approach to the screening of CS in bone clinics. Although LNSC measurement could be preferred for its high sensitivity as first diagnostic approach in patients with otherwise asymptomatic hypercortisolism, performing DST appears to be more reasonable also due to the still limited routine availability for salivary cortisol assays. Additional tests could be performed to confirm/refine CS diagnosis in case of inadequate suppression of cortisol after dexamethasone.

Patients with densitomtric osteoporosis or vertebral clinical or morphometric fractures at high risk of having CS due to concomitant classical manifestations of the disease should undergo to all first level tests for confirming/excluding hypercortisolism.

#### Bone tests

So far, the bone health approach opportunistically used for screening CS included bone densitometry with DXA and specifically those patients resulting in the osteoporotic range after the DXA test [8] or the evaluation of patients undergoing clinical fragility fractures [35]. However, excess glucocorticoids highly increase the risk of fragility fractures [72] often with normal or osteopenic results at DXA [73] and clinical diagnosis largely underestimate vertebral fractures which are the landmark of bone involvement in CS [74]. Therefore, it is highly likely that currently used bone screening methods only in part may have the potential to bring to clinical attention all patients candidates to biochemical assessment for CS. We suggest that widespread use of additional DXA tools such as morphometry and TBS quality assessment should be able to optimize the role of bone clinic in CS screening eventually increasing the already quite high numbers reported further corroborating the idea that bone tests may be a cost/effective method to screen for CS and allow an early tumor- and/or bone- targeted treatment [75–77] the efficacy of which has been suggested [78–81] but not yet proven [42].

## Conclusion

A rationale does exist for screening for CS patients attending bone centers. In fact, an effective screening would likely be able to reduce the diagnostic delay which is one of the unmet needs in management of endogenous hypercortisolism [7]. However, besides single center limited studies no such an extensive screening was already perfomed in large multicenter series either in pituitary- or adrenal-driven CS [25]. Due to the lack of such studies, it is likely that selective screenings in specifically pre-determined high risk patients would be more cost/effective and feasible in the screening of CS in bone centers. However, also in this latter case multicenter large-scale studies are still needed.

## References

[1] Canalis E, Mazziotti G, Giustina A, Bilezikian JP (2007) Glucocorticoid-induced osteoporosis: pathophysiology and therapy. Osteoporos Int 18(10):1319–1328. 10.1007/s00198-007-0394-0 17566815 10.1007/s00198-007-0394-0

[2] Fleseriu M, Auchus R, Bancos I, Ben-Shlomo A, Bertherat J, Biermasz NR, Boguszewski CL, Bronstein MD, Buchfelder M, Carmichael JD, Casanueva FF, Castinetti F, Chanson P, Findling J, Gadelha M, Geer EB, Giustina A, Grossman A, Gurnell M, Ho K, Ioachimescu AG, Kaiser UB, Karavitaki N, Katznelson L, Kelly DF, Lacroix A, McCormack A, Melmed S, Molitch M, Mortini P, Newell-Price J, Nieman L, Pereira AM, Petersenn S, Pivonello R, Raff H, Reincke M, Salvatori R, Scaroni C, Shimon I, Stratakis CA, Swearingen B, Tabarin A, Takahashi Y, Theodoropoulou M, Tsagarakis S, Valassi E, Varlamov EV, Vila G, Wass J, Webb SM, Zatelli MC, Biller BMK (2021) Consensus on diagnosis and management of Cushing’s disease: a guideline update. Lancet Diabetes Endocrinol 9(12):847–875. 10.1016/S2213-8587(21)00235-7 34687601 10.1016/S2213-8587(21)00235-7 PMC8743006

[3] Frara S, Allora A, di Filippo L, Formenti AM, Loli P, Polizzi E, Tradati D, Ulivieri FM, Giustina A (2021) Osteopathy in mild adrenal Cushing’s syndrome and Cushing disease. Best Pract Res Clin Endocrinol Metab 35(2):101515. 10.1016/j.beem.2021.101515 33795196 10.1016/j.beem.2021.101515

[4] Frara S, Melin Uygur M, di Filippo L, Doga M, Losa M, Santoro S, Mortini P, Giustina A (2022) High prevalence of vertebral fractures Associated with preoperative GH levels in patients with recent diagnosis of Acromegaly. J Clin Endocrinol Metab 107(7):e2843–e2850. 10.1210/clinem/dgac183 35349698 10.1210/clinem/dgac183

[5] Arnaldi G, Martino M (2019) Androgens in Cushing’s syndrome. Front Horm Res 53:77–91. 10.1159/000494904 31499501 10.1159/000494904

[6] Arnaldi G, Angeli A, Atkinson AB, Bertagna X, Cavagnini F, Chrousos GP, Fava GA, Findling JW, Gaillard RC, Grossman AB, Kola B, Lacroix A, Mancini T, Mantero F, Newell-Price J, Nieman LK, Sonino N, Vance ML, Giustina A, Boscaro M (2003) Diagnosis and complications of Cushing’s syndrome: a consensus statement. J Clin Endocrinol Metab 88(12):5593–5602. 10.1210/jc.2003-030871 14671138 10.1210/jc.2003-030871

[7] Pivonello R, Scaroni C, Polistena B, Migliore A, Giustina A (2023) Unmet needs on the current medical management of Cushing’s syndrome: results from a Delphi panel of Italian endocrinologists. J Endocrinol Invest 19:1–12. 10.1007/s40618-023-02058-8 10.1007/s40618-023-02058-8 PMC10115381 37076758

[8] Chiodini I, Mascia ML, Muscarella S, Battista C, Minisola S, Arosio M, Santini SA, Guglielmi G, Carnevale V, Scillitani A (2007) Subclinical hypercortisolism among outpatients referred for osteoporosis. Ann Intern Med 16(8):541–548. 10.7326/0003-4819-147-8-200710160-00006 10.7326/0003-4819-147-8-200710160-00006 17938392

[9] Canalis E, Giustina A (2001) Glucocorticoid-induced osteoporosis: summary of a workshop. J Clin Endocrinol Metab 86(12):5681–5685. 10.1210/jcem.86.12.8066 11739419 10.1210/jcem.86.12.8066

[10] Chotiyarnwong P, McCloskey EV (2020) Pathogenesis of glucocorticoid-induced osteoporosis and options for treatment. Nat Rev Endocrinol 16(8):437–447. 10.1038/s41574-020-0341-0 32286516 10.1038/s41574-020-0341-0

[11] Manelli F, Giustina A (2000) Glucocorticoid-induced osteoporosis. Trends Endocrinol Metab 11(3):79–85. 10.1016/s1043-2760(00)00234-4 10707047 10.1016/s1043-2760(00)00234-4

[12] Canalis E, Bilezikian JP, Angeli A, Giustina A (2004) Perspectives on glucocorticoid-induced osteoporosis. Bone 34(4):593–598. 10.1016/j.bone.2003.11.026 15050888 10.1016/j.bone.2003.11.026

[13] Mazziotti G, Giustina A (2013) Glucocorticoids and the regulation of growth hormone secretion. Nat Rev Endocrinol 9(5):265–276. 10.1038/nrendo.2013.5 23381030 10.1038/nrendo.2013.5

[14] Formenti AM, Maffezzoni F, Doga M, Mazziotti G, Giustina A (2017) Growth hormone deficiency in treated acromegaly and active Cushing’s syndrome. Best Pract Res Clin Endocrinol Metab 31(1):79–90. 10.1016/j.beem.2017.03.002 28477735 10.1016/j.beem.2017.03.002

[15] Mazziotti G, Formenti AM, Adler RA, Bilezikian JP, Grossman A, Sbardella E, Minisola S, Giustina A (2016) Glucocorticoid-induced osteoporosis: pathophysiological role of GH/IGF-I and PTH/VITAMIN D axes, treatment options and guidelines. Endocrine 54(3):603–611. 10.1007/s12020-016-1146-8 27766553 10.1007/s12020-016-1146-8

[16] Skversky AL, Kumar J, Abramowitz MK, Kaskel FJ, Melamed ML (2011) Association of glucocorticoid use and low 25-hydroxyvitamin D levels: results from the National Health and Nutrition Examination Survey (NHANES): 2001–2006. J Clin Endocrinol Metab 96(12):3838–3845. 10.1210/jc.2011-1600 21956424 10.1210/jc.2011-1600 PMC3232615

[17] Bilezikian JP, Formenti AM, Adler RA, Binkley N, Bouillon R, Lazaretti-Castro M, Marcocci C, Napoli N, Rizzoli R, Giustina A (2021) Vitamin D: Dosing, levels, form, and route of administration: does one approach fit all? Rev Endocr Metab Disord 22(4):1201–1218. 10.1007/s11154-021-09693-7 34940947 10.1007/s11154-021-09693-7 PMC8696970

[18] Bonadonna S, Burattin A, Nuzzo M, Bugari G, Rosei EA, Valle D, Iori N, Bilezikian JP, Veldhuis JD, Giustina A (2005) Chronic glucocorticoid treatment alters spontaneous pulsatile parathyroid hormone secretory dynamics in human subjects. Eur J Endocrinol 152(2):199–205. 10.1530/eje.1.01841 15745926 10.1530/eje.1.01841

[19] Donini LM, Busetto L, Bischoff SC, Cederholm T, Ballesteros-Pomar MD, Batsis JA, Bauer JM, Boirie Y, Cruz-Jentoft AJ, Dicker D, Frara S, Frühbeck G, Genton L, Gepner Y, Giustina A, Gonzalez MC, Han HS, Heymsfield SB, Higashiguchi T, Laviano A, Lenzi A, Nyulasi I, Parrinello E, Poggiogalle E, Prado CM, Salvador J, Rolland Y, Santini F, Serlie MJ, Shi H, Sieber CC, Siervo M, Vettor R, Villareal DT, Volkert D, Yu J, Zamboni M, Barazzoni R (2022) Definition and diagnostic criteria for sarcopenic obesity: ESPEN and EASO consensus statement. Clin Nutr 41(4):990–1000. 10.1016/j.clnu.2021.11.014 35227529 10.1016/j.clnu.2021.11.014

[20] Mazziotti G, Bilezikian J, Canalis E, Cocchi D, Giustina A (2012) New understanding and treatments for osteoporosis. Endocrine 41(1):58–69. 10.1007/s12020-011-9570-2 22180055 10.1007/s12020-011-9570-2

[21] Mancini T, Doga M, Mazziotti G, Giustina A (2004) Cushing’s syndrome and bone. Pituitary 7(4):249–252. 10.1007/s11102-005-1051-2 16010458 10.1007/s11102-005-1051-2

[22] Tauchmanovà L, Pivonello R, De Martino MC, Rusciano A, De Leo M, Ruosi C, Mainolfi C, Lombardi G, Salvatore M, Colao A (2007) Effects of sex steroids on bone in women with subclinical or overt endogenous hypercortisolism. Eur J Endocrinol 157:359–366. 10.1530/EJE-07-0137 17766720 10.1530/EJE-07-0137

[23] Trementino L, Appolloni G, Ceccoli L, Marcelli G, Concettoni C, Boscaro M, Arnaldi G (2014) Bone complications in patients with Cushing’s syndrome: looking for clinical, biochemical, and genetic determinants. Osteoporos Int 25:913–921. 10.1007/s00198-013-2520-5 24126765 10.1007/s00198-013-2520-5

[24] Zilio M, Barbot M, Ceccato F, Camozzi V, Bilora F, Casonato A, Frigo AC, Albiger N, Daidone V, Mazzai L, Mantero F, Scaroni C (2014) Diagnosis and complications of Cushing’s disease: gender-related differences. Clin Endocrinol (Oxf) 80(3):403–410. 10.1111/cen.12299 23889360 10.1111/cen.12299

[25] Chiodini I, Vainicher CE, Morelli V, Palmieri S, Cairoli E, Salcuni AS, Copetti M, Scillitani A (2016) MECHANISMS IN ENDOCRINOLOGY: endogenous subclinical hypercortisolism and bone: a clinical review. Eur J Endocrinol 175(6):R265–R282. 10.1530/EJE-16-0289 27412441 10.1530/EJE-16-0289

[26] dos Santos CV, Vieira Neto L, Madeira M, Alves Coelho MC, de Mendonça LM, Paranhos-Neto Fde P, Lima IC, Gadelha MR, Farias ML (2015) Bone density and microarchitecture in endogenous hypercortisolism. Clin Endocrinol (Oxf) 83(4):468–474. 10.1111/cen.12812 25940452 10.1111/cen.12812

[27] Apaydın T, Yavuz DG (2021) Assessment of non-traumatic vertebral fractures in Cushing’s syndrome patients. J Endocrinol Invest 44(8):1767–1773. 10.1007/s40618-020-01496-y 33420960 10.1007/s40618-020-01496-y

[28] Chiodini I, Carnevale V, Torlontano M, Fusilli S, Guglielmi G, Pileri M, Modoni S, Di Giorgio A, Liuzzi A, Minisola S, Cammisa M, Trischitta V, Scillitani A (1998) Alterations of bone turnover and bone mass at different skeletal sites due to pure glucocorticoid excess: study in eumenorrheic patients with Cushing’s syndrome. J Clin Endocrinol Metab 83(6):1863–1867. 10.1210/jcem.83.6.4880 9626110 10.1210/jcem.83.6.4880

[29] Uygur MM, Frara S, di Filippo L, Giustina A (2023) New tools for bone health assessment in secreting pituitary adenomas. Trends Endocrinol Metab 34(4):231–242. 10.1016/j.tem.2023.01.006 36869001 10.1016/j.tem.2023.01.006

[30] Belaya ZE, Hans D, Rozhinskaya LY, Dragunova NV, Sasonova NI, Solodovnikov AG, Tsoriev TT, Dzeranova LK, Melnichenko GA, Dedov II (2015) The risk factors for fractures and trabecular bone-score value in patients with endogenous Cushing’s syndrome. Arch Osteoporos 10:44. 10.1007/s11657-015-0244-1 26608406 10.1007/s11657-015-0244-1

[31] Godang K, Ueland T, Bollerslev J (1999) Decreased bone area, bone mineral content, formative markers, and increased bone resorptive markers in endogenous Cushing’s syndrome. Eur J Endocrinol 141(2):126–131. 10.1530/eje.0.1410126 10427155 10.1530/eje.0.1410126

[32] Mazziotti G, Frara S, Giustina A (2018) Pituitary diseases and Bone. Endocr Rev 39(4):440–488. 10.1210/er.2018-00005 29684108 10.1210/er.2018-00005

[33] Formenti AM, Dalla Volta A, di Filippo L, Berruti A, Giustina A (2021) Effects of Medical treatment of prostate Cancer on Bone Health. Trends Endocrinol Metab 32(3):135–158. 10.1016/j.tem.2020.12.004 33509658 10.1016/j.tem.2020.12.004

[34] di Filippo L, Formenti AM, Doga M, Pedone E, Rovere-Querini P, Giustina A (2021) Radiological thoracic vertebral fractures are highly prevalent in COVID-19 and predict Disease outcomes. J Clin Endocrinol Metab 106(2):e602–e614. 10.1210/clinem/dgaa738 33159451 10.1210/clinem/dgaa738 PMC7797741

[35] Mancini T, Mazziotti G, Doga M, Carpinteri R, Simetovic N, Vescovi PP, Giustina A (2009) Vertebral fractures in males with type 2 diabetes treated with rosiglitazone. Bone 45(4):784–788. 10.1016/j.bone.2009.06.006 19527806 10.1016/j.bone.2009.06.006

[36] Tauchmanovà L, Pivonello R, Di Somma C, Rossi R, De Martino MC, Camera L, Klain M, Salvatore M, Lombardi G, Colao A (2006) Bone demineralization and vertebral fractures in endogenous cortisol excess: role of disease etiology and gonadal status. J Clin Endocrinol Metab 91(5):1779–1784. 10.1210/jc.2005-0582 16522701 10.1210/jc.2005-0582

[37] Zavatta G, Vicennati V, Altieri P, Tucci L, Colombin G, Coscia K, Mosconi C, Balacchi C, Fanelli F, Malagrinò M, Magagnoli M, Golfieri R, Pagotto U, Di Dalmazi G (2023) Mild autonomous cortisol secretion in adrenal incidentalomas and risk of fragility fractures: a large cross-sectional study. Eur J Endocrinol 188(4):343–352. 10.1093/ejendo/lvad038 36952249 10.1093/ejendo/lvad038

[38] Frara S, di Filippo L, Doga M, Loli P, Casanueva FF, Giustina A (2022) Novel approaches to bone comorbidity in Cushing’s disease: an update. Pituitary 25(5):754–759. 10.1007/s11102-022-01252-w 35849272 10.1007/s11102-022-01252-w

[39] Braun LT, Vogel F, Zopp S, Marchant Seiter T, Rubinstein G, Berr CM, Künzel H, Beuschlein F, Reincke M (2022) Whom should we screen for Cushing Syndrome? The Endocrine Society Practice Guideline recommendations 2008 Revisited. J Clin Endocrinol Metab 18(9):e3723–e3730. 10.1210/clinem/dgac379 10.1210/clinem/dgac379 PMC9387700 35730067

[40] Chiodini I, Guglielmi G, Battista C, Carnevale V, Torlontano M, Cammisa M, Trischitta V, Scillitani A (2004) Spinal volumetric bone mineral density and vertebral fractures in female patients with adrenal incidentalomas: the effects of subclinical hypercortisolism and gonadal status. J Clin Endocrinol Metab 89(5):2237–2241. 10.1210/jc.2003-031413 15126547 10.1210/jc.2003-031413

[41] Pugliese F, Salcuni AS, Battista C, Carnevale V, Guglielmi G, Columbu C, Velluzzi F, Giovanelli L, Eller-Vainicher C, Scillitani A, Chiodini (2021) Prevalence of less severe hypercortisolism in fractured patients admitted in an outpatient clinic for metabolic bone diseases. Endocrine 73(1):203–208. 10.1007/s12020-021-02616-1 33484412 10.1007/s12020-021-02616-1

[42] Nieman LK (2007) Screening for reversible osteoporosis: is cortisol a culprit? Ann Intern Med 16;147(8):582–584. 10.7326/0003-4819-147-8-200710160-00012 10.7326/0003-4819-147-8-200710160-00012 17938397

[43] Nieman LK, Biller BM, Findling JW, Newell-Price J, Savage MO, Stewart PM, Montori VM (2008) The diagnosis of Cushing’s syndrome: an endocrine Society Clinical Practice Guideline. J Clin Endocrinol Metab 93(5):1526–1540. 10.1210/jc.2008-0125 18334580 10.1210/jc.2008-0125 PMC2386281

[44] Wilson JM, Jungner G (1968) Principles and practice of screening for disease. World Health Organization, GENEVA. (Public Health Paper NUMBER 34.)https://apps.who.int/iris/handle/10665/37650

[45] Chiodini I, Torlontano M, Carnevale V, Trischitta V, Scillitani A (2008) Skeletal involvement in adult patients with endogenous hypercortisolism. J Endocrinol Invest 31:267–276. 10.1007/BF03345601 18401211 10.1007/BF03345601

[46] Shimon I (2015) Screening for Cushing’s syndrome: is it worthwhile? Pituitary 18(2):201–205. 10.1007/s11102-015-0634-9 25578150 10.1007/s11102-015-0634-9

[47] Trementino L, Ceccoli L, Concettoni C, Marcelli G, Michetti G, Boscaro M, Arnaldi G (2014) Fracture risk assessment before and after resolution of endogenous hypercortisolism: is the FRAX^®^ algorithm useful? J Endocrinol Invest 37:957–965. 10.1007/s40618-014-0126-1 25012159 10.1007/s40618-014-0126-1

[48] Formenti AM, Tecilazich F, Giubbini R, Giustina A (2019) Risk of vertebral fractures in hypoparathyroidism. Rev Endocr Metab Disord 20(3):295–302. 10.1007/s11154-019-09507-x 31471845 10.1007/s11154-019-09507-x

[49] Newell-Price J, Bertagna X, Grossman AB, Nieman LK (2006) Cushing’s syndrome. Lancet 367:1605–1617. 10.1016/S0140-6736(06)68699-6 16698415 10.1016/S0140-6736(06)68699-6

[50] Galm BP, Qiao N, Klibanski A, Biller BMK, Tritos NA (2020) Accuracy of Laboratory tests for the diagnosis of Cushing Syndrome. J Clin Endocrinol Metab 105(6):2081–2094. 10.1210/clinem/dgaa105 10.1210/clinem/dgaa105 32133504

[51] Parasiliti-Caprino M, Bioletto F, Frigerio T, D’Angelo V, Ceccato F, Ferraù F, Ferrigno R, Minnetti M, Scaroni C, Cannavò S, Pivonello R, Isidori A, Broglio F, Giordano R, Spinello M, Grottoli S, Arvat E (2021) A New Clinical Model to Estimate the Pre-test Probability of Cushing’s syndrome: the Cushing score. Front Endocrinol (Lausanne) 5:12:747549. 10.3389/fendo.2021.747549 10.3389/fendo.2021.747549 PMC8524092 34675882

[52] Giustina A, Uygur MM, Frara S, Barkan A, Biermasz NR, Chanson P, Freda P, Gadelha M, Kaiser UB, Lamberts S, Laws E, Nachtigall LB, Popovic V, Reincke M, Strasburger C, van der Lely AJ, Wass JAH, Melmed S, Casanueva FF (2023) Pilot study to define criteria for Pituitary Tumors Centers of Excellence (PTCOE): results of an audit of leading international centers. Pituitary 26(5):583–596. 10.1007/s11102-023-01345-0 37640885 10.1007/s11102-023-01345-0 PMC10539196

[53] Braun LT, Riester A, Oßwald-Kopp A, Fazel J, Rubinstein G, Bidlingmaier M, Beuschlein F, Reincke M (2019) Toward a diagnostic score in Cushing’s syndrome. Front Endocrinol (Lausanne) 10:766. 10.3389/fendo.2019.00766 31787931 10.3389/fendo.2019.00766 PMC6856055

[54] Ponzetto F, Settanni F, Parasiliti-Caprino M, Rumbolo F, Nonnato A, Ricciardo M, Amante E, Priolo G, Vitali S, Anfossi L, Arvat E, Ghigo E, Giordano R, Mengozzi G (2020) Reference ranges of late-night salivary cortisol and cortisone measured by LC-MS/MS and accuracy for the diagnosis of Cushing’s syndrome. J Endocrinol Invest 43(12):1797–1806. 10.1007/s40618-020-01388-1 32772255 10.1007/s40618-020-01388-1

[55] Hansen AM, Garde AH, Persson R (2008) Sources of biological and methodological variation in salivary cortisol and their impact on measurement among healthy adults: a review. Scand J Clin Lab Invest 68(6):448–458. 10.1080/00365510701819127 18609093 10.1080/00365510701819127

[56] Fassnacht M, Tsagarakis S, Terzolo M, Tabarin A, Sahdev A, Newell-Price J, Pelsma I, Marina L, Lorenz K, Bancos I, Arlt W, Dekkers OM (2023) European Society of Endocrinology clinical practice guidelines on the management of adrenal incidentalomas, in collaboration with the European Network for the study of adrenal tumors. Eur J Endocrinol 189(1):G1–G42. 10.1093/ejendo/lvad066 37318239 10.1093/ejendo/lvad066

[57] Ceccato F, Artusi C, Barbot M, Lizzul L, Pinelli S, Costantini G, Niero S, Antonelli G, Plebani M, Scaroni C (2020) Dexamethasone measurement during low-dose suppression test for suspected hypercortisolism: threshold development with and validation. J Endocrinol Invest 43(8):1105–1113. 10.1007/s40618-020-01197-6 32060745 10.1007/s40618-020-01197-6

[58] Palmieri S, Morelli V, Polledri E, Fustinoni S, Mercadante R, Olgiati L, Eller Vainicher C, Cairoli E, Zhukouskaya VV, Beck-Peccoz P, Chiodini I (2013) The role of salivary cortisol measured by liquid chromatography-tandem mass spectrometry in the diagnosis of subclinical hypercortisolism. Eur J Endocrinol 168(3):289–296. 10.1530 23211572 10.1530/EJE-12-0803

[59] Ceccato F, Antonelli G, Frigo AC, Regazzo D, Plebani M, Boscaro M, Scaroni C (2017) First-line screening tests for Cushing’s syndrome in patients with adrenal incidentaloma: the role of urinary free cortisol measured by LC-MS/MS. J Endocrinol Invest 40(7):753–760. 10.1007/s40618-017-0644-8 28247215 10.1007/s40618-017-0644-8

[60] Petersenn S (2021) Biochemical diagnosis of Cushing’s disease: screening and confirmatory testing. Best Pract Res Clin Endocrinol Metab 35(1):101519. 10.1016/j.beem.2021.101519 33757676 10.1016/j.beem.2021.101519

[61] Mazziotti G, Delgado A, Maffezzoni F, Formenti A, Giustina A (2016) Skeletal fragility in endogenous hypercortisolism. Front Horm Res 46:66–73. 10.1159/000443866 27210111 10.1159/000443866

[62] Dekkers OM, Horváth-Puhó E, Jørgensen JO, Cannegieter SC, Ehrenstein V, Vandenbroucke JP, Pereira AM, Sørensen HT (2013) Multisystem morbidity and mortality in Cushing’s syndrome: a cohort study. J Clin Endocrinol Metab 98:2277–2284. 10.1210/jc.2012-3582 23533241 10.1210/jc.2012-3582

[63] Mancini T, Porcelli T, Giustina A (2010) Treatment of cushing disease: overview and recent findings. Ther Clin Risk Manag 6:505–516. 10.2147/TCRM.S12952 21063461 10.2147/TCRM.S12952 PMC2963160

[64] Omura M, Saito J, Yamaguchi K, Kakuta Y, Nishikawa T (2004) Prospective study on the prevalence of secondary hypertension among hypertensive patients visiting a general outpatient clinic in Japan. Hypertens Res 27:193–202. 10.1291/hypres.27.193 15080378 10.1291/hypres.27.193

[65] Mazziotti G, Formenti AM, Frara S, Maffezzoni F, Doga M, Giustina A (2017) Diabetes in Cushing Disease. Curr Diab Rep 17(5):32. 10.1007/s11892-017-0860-9 28364356 10.1007/s11892-017-0860-9

[66] Vrkljan M, Vrkljan M, Thaller V, Lovricević I, Gaćina P, Resetić J, Bekić M, Sonicki Z (2001) Depressive disorder as possible risk factor of osteoporosis. Coll Antropol 25:485–492 11811278

[67] Melmed S, Kaiser UB, Lopes MB, Bertherat J, Syro LV, Raverot G, Reincke M, Johannsson G, Beckers A, Fleseriu M, Giustina A, Wass JAH, Ho KKY (2022) Clinical Biology of the Pituitary Adenoma. Endocr Rev 43(6):1003–1037. 10.1210/endrev/bnac010 35395078 10.1210/endrev/bnac010 PMC9695123

[68] Mondin A, Barbot M, Voltan G, Tizianel I, Vedolin CK, Mazzeo P, Lazzara M, Boscaro M, Scaroni C, Ceccato F (2023) Second-line tests in the differential diagnosis of neoplastic and non-neoplastic hypercortisolism: a systematic review and meta-analysis. J Endocrinol Invest 46(10):1947–1959. 10.1007/s40618-023-02099-z 37079177 10.1007/s40618-023-02099-z PMC10514124

[69] Frara S, Rodriguez-Carnero G, Formenti AM, Martinez-Olmos MA, Giustina A, Casanueva FF (2020) Pituitary Tumors Centers of Excellence. Endocrinol Metab Clin North Am 49(3):553–564. 10.1016/j.ecl.2020.05.010 32741488 10.1016/j.ecl.2020.05.010

[70] Couselo M, Frara S, Giustina A, Casanueva FF (2022) Pituitary tumor centers of excellence for Cushing’s disease. Pituitary 25(5):772–775. 10.1007/s11102-022-01264-6 36087228 10.1007/s11102-022-01264-6 PMC9587956

[71] Pivonello R, Isidori AM, De Martino MC, Newell-Price J, Biller BM, Colao A (2016) Complications of Cushing’s syndrome: state of the art. Lancet Diabetes Endocrinol 4(7):611–629. 10.1016/S2213-8587(16)00086-3 27177728 10.1016/S2213-8587(16)00086-3

[72] Angeli A, Guglielmi G, Dovio A, Capelli G, de Feo D, Giannini S, Giorgino R, Moro L, Giustina A (2006) High prevalence of asymptomatic vertebral fractures in post-menopausal women receiving chronic glucocorticoid therapy: a cross-sectional outpatient study. Bone 39(2):253–259. 10.1016/j.bone.2006.02.005 16574519 10.1016/j.bone.2006.02.005

[73] Minetto M, Reimondo G, Osella G, Ventura M, Angeli A, Terzolo M (2004) Bone loss is more severe in primary adrenal than in pituitary-dependent Cushing’s sindrome. Osteoporos Int 15:855–874. 10.1007/s00198-004-1616-3 15034643 10.1007/s00198-004-1616-3

[74] Vestergaard P, Lindholm J, Jørgensen JO, Hagen C, Hoeck HC, Laurberg P, Rejnmark L, Brixen K, Kristensen LØ, Feldt-Rasmussen U, Mosekilde L (2002) Increased risk of osteoporotic fractures in patients with Cushing’s syndrome. Eur J Endocrinol 146:51–56. 10.1530/eje.0.1460051 11751067 10.1530/eje.0.1460051

[75] Fleseriu M, Castinetti F, Gadelha M, Giustina A, Lacroix A, Melmed S, Newell-Price J, Pivonello R, Reincke M, Biller BMK (2022) Osilodrostat for the treatment of Cushing’s disease: efficacy, stability, and persistence - authors’ reply. Lancet Diabetes Endocrinol 10(6):385–387. 10.1016/S2213-8587(22)00135-8 35597255 10.1016/S2213-8587(22)00135-8

[76] Scillitani A, Mazziotti G, Di Somma C, Moretti S, Stigliano A, Pivonello R, Giustina A, Colao A, ABC Group (2014) Treatment of skeletal impairment in patients with endogenous hypercortisolism: when and how? Osteoporos Int 25(2):441–446. 10.1007/s00198-013-2588-y 24311114 10.1007/s00198-013-2588-y

[77] Di Somma C, Colao A, Pivonello R, Klain M, Faggiano A, Tripodi FS, Merola B, Salvatore M, Lombardi G (1998) Effectiveness of chronic treatment with alendronate in the osteoporosis of Cushing’s disease. Clin Endocrinol (Oxf) 48(5):655–662. 10.1046/j.1365-2265.1998.00486.x 9666879 10.1046/j.1365-2265.1998.00486.x

[78] Di Somma C, Pivonello R, Loche S, Faggiano A, Klain M, Salvatore M, Lombardi G, Colao A (2003) Effect of 2 years of cortisol normalization on the impaired bone mass and turnover in adolescent and adult patients with Cushing’s disease: a prospective study. Clin Endocrinol (Oxf) 58:302–308. 10.1046/j.1365-2265.2003.01713.x 12608935 10.1046/j.1365-2265.2003.01713.x

[79] Faggiano A, Pivonello R, Filippella M, Di Somma C, Orio F Jr, Lombard G, Colao A (2001) Spine abnormalities and damage in patients cured from Cushing’s disease. Pituitary 4(3):153–161. 10.1023/a:1015362822901 12138988 10.1023/a:1015362822901

[80] Futo L, Toke J, Patócs A, Szappanos A, Varga I, Gláz E, Tulassay Z, Rácz K, Tóth M (2008) Skeletal differences in bone mineral area and content before and after cure of endogenous Cushing’s syndrome. Osteoporos Int 19(7):941–949. 10.1007/s00198-007-0514-x 18043854 10.1007/s00198-007-0514-x

[81] Randazzo ME, grossrubatscher EM, Dalino Ciaramella P, Vanzulli A, Loli P (2012) Spontaneous recovery of bone mass after cure of endogenous hypercortisolism. Pituitary 15(2):193–201. 10.1007/s11102-011-0306-3 21476062 10.1007/s11102-011-0306-3

