# Correction: Screening for endogenous hypercortisolism in patients with osteoporosis and fractures: why, when and how

**DOI:** 10.1007/s40618-024-02488-y

**Published:** 2024-11-09

**Authors:** Roberta Giordano, Mirko Parasiliti Caprino, Paola Loli, Andrea Giustina

**Affiliations:** 1 Department of Biological and Clinical Sciences, University of Turin, Turin, Italy; 2 Division of Endocrinology, Diabetology and Metabolism, Department of Medical Sciences, University of Turin, Turin, Italy; 3 Institute of Endocrine and Metabolic Sciences (IEMS), Università Vita-Salute San Raffaele IRCCS Ospedale San Raffaele, Via Olgettina, 58, Milano, 20132 Italy

**Correction:** Journal of Endocrinological Investigation 10.1007/s40618-024-02450-y

Roberta Giordano1 · Mirko Parasiliti Caprino2 · Paola Loli3 · Andrea Giustina3

In the original version of this article, Some part of the Funding information was missing. The funding information was previously read "The authors did not receive support from any organization for the submitted work. Recordati Rare Diseases supported open acpublication of the collection" should have been "The authors did not receive support from any organization for the submitted work. Recordati Rare Diseases supported open acpublication of the collection - Open Access funding for this article was provided by Recordati Rare Diseases Italy S.r.l. The funder had no role in the preparation, review, or approval of the manuscript".

The original article has been corrected.

Roberta Giordano

ORCID: http://orcid.org/0000-0001-8713-3532

Department of Biological and Clinical Sciences, University of Turin, Turin, Italy

Mirko Parasiliti Caprino

ORCID: http://orcid.org/0000-0002-6930-7073

Division of Endocrinology, Diabetology and Metabolism, Department of Medical Sciences, University of Turin, Turin, Italy

Paola Loli

ORCID:http://orcid.org/0000-0002-5545-550X

Institute of Endocrine and Metabolic Sciences (IEMS), Università Vita-Salute San Raffaele IRCCS Ospedale San Raffaele, Via Olgettina, 58, Milano, 20,132, Italy

Andrea Giustina

ORCID: http://orcid.org/0000-0001-6783-3398

giustina.andrea@hsr.it

Institute of Endocrine and Metabolic Sciences (IEMS), Università Vita-Salute San Raffaele IRCCS Ospedale San Raffaele, Via Olgettina, 58, Milano, 20,132, Italy

